# Neurotrophins in the Neuropathophysiology, Course, and Complications of Obstructive Sleep Apnea—A Narrative Review

**DOI:** 10.3390/ijms24031808

**Published:** 2023-01-17

**Authors:** Agata Gabryelska, Szymon Turkiewicz, Marta Ditmer, Marcin Sochal

**Affiliations:** Department of Sleep Medicine and Metabolic Disorders, Medical University of Lodz, 90-419 Lodz, Poland

**Keywords:** BDNF, OSA, neurotrophins, sleep, PSG

## Abstract

Obstructive sleep apnea (OSA) is a disorder characterized by chronic intermittent hypoxia and sleep fragmentation due to recurring airway collapse during sleep. It is highly prevalent in modern societies, and due to its pleiotropic influence on the organism and numerous sequelae, it burdens patients and physicians. Neurotrophins (NTs), proteins that modulate the functioning and development of the central nervous system, such as brain-derived neurotrophic factor (BDNF), have been associated with OSA, primarily due to their probable involvement in offsetting the decline in cognitive functions which accompanies OSA. However, NTs influence multiple aspects of biological functioning, such as immunity. Thus, extensive evaluation of their role in OSA might enlighten the mechanism behind some of its elusive features, such as the increased risk of developing an immune-mediated disease or the association of OSA with cardiovascular diseases. In this review, we examine the interactions between NTs and OSA and discuss their contribution to OSA pathophysiology, complications, as well as comorbidities.

## 1. Introduction

**Figure 1:**
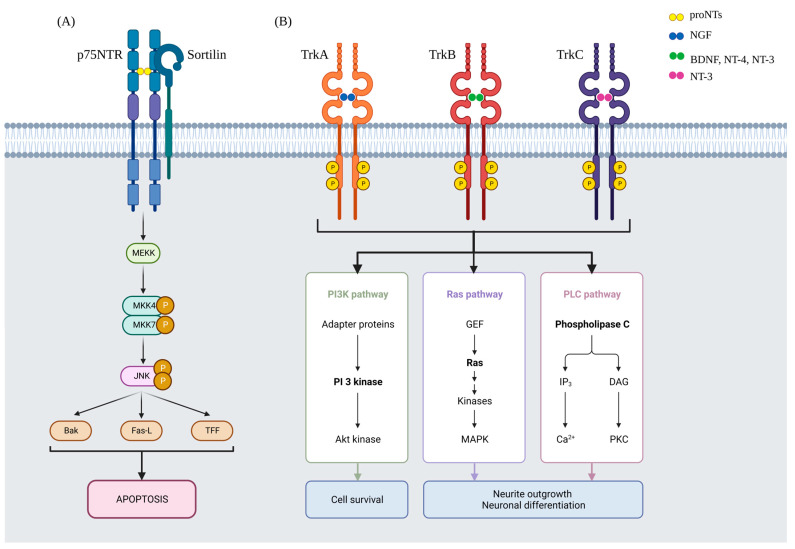
Neurotrophins pathways. (**A**) proNTs, including proBDNF and proNGF (proNT-3, proNT-4 in a lesser extent), have pro-apoptotic activity. They make a complex with sortilin and p75NTR, initiating the JNK pathway. (**B**) Trk family are receptor tyrosine kinases family which shows affinity to NTs. They act through many pathways, such as PI3K/AKT, Ras/MAPK, and PLC/PKC. Akt—serine/threonine protein kinase; Bak—BCL-2 homologous antagonist/killer; BDNF—brain-derived neurotrophic factor; DAG—diacyloglycerol; Fas-L—tumor necrosis factor ligand superfamily member 6; IP3—inositol trisphosphate; JNK—c-Jun N-terminal kinase; MAPK—mitogen-activated protein kinase; MEKK—mitogen-activated protein kinase kinase kinase 1; MKK4—mitogen-activated protein kinase kinase 4; MKK7—mitogen-activated protein kinase kinase 7; NGF—nerve growth factor; NT-3—neurotrophin 3; NT-4—neurotrophin 4; p75NTR—p75 neurotrophic receptor; PI3K—PI3 -kinase type 3; PKC—protein kinase C; proNTs—premature neurotrophins; Ras—KRAS proto-oncogen; TFF—trefoil factor 1; TrkA/B/C—tropomyosin receptor kinase A/B/C.

Neurotrophins (NTs) are proteins regulating the development and functioning of the nervous system. There are four canonical neurotrophins: nerve growth factor (NGF), brain-derived neurotrophic factor (BDNF), neurotrophin-3 (NT-3), and neurotrophin-4 (NT-4), which share similar tertiary structures. There are also neurotrophic factors, such as glial cell-line-derived neurotrophic factor (GDNF), which functionally, but not structurally, resemble the aforementioned NTs; however, the terms neurotrophic factor and neurotrophin can be used interchangeably. NTs work through the tropomyosin receptor kinase (Trk) family, which are receptor tyrosine kinases. There are three isoforms—TrkA, TrkB, and TrkC. TrkA shows affinity primarily to NGF, TrkC to NT-3, then TrkB binds BDNF, NT-3, and NT4 [1,2,3,4]. Activation of the receptors initiates a cascade of complex signaling pathways, including Ras/MAPK, PI3K/Akt, and PKC/PLC (Figure 1B) [5]. They further neuronal cell survival, differentiation, and the outgrowth of the neurites [5]. All of the NTs are synthesized in premature forms (proNTs). proNTs have oppositional action to the mature equivalent, leading to cell degeneration and neuronal apoptosis. proNTs bind to sortilin, which allows the activation of the p75NTR [5]. Its pathway provides activation of JNK and pro-apoptotic factors, such as Bak and Fas-L (Figure 1A) [6,7].

NTs are indispensable in the process of neuro- and gliogenesis. It has been proven that neural stem cells constitutively secrete, among others, NGF and BDNF [8]. Neurogenesis involves the formation of new neurons from neural precursor cells; neurogenesis generally occurs most intensely during embryonal development and two years after birth [9]. However, it might persist well into adulthood in specific brain regions, known as neurogenic niches, such as the dentate gyrus in the hippocampus [9,10]. Gliogenesis is a related process, producing non-neuronal cells of the nervous system—astrocytes, oligodendrocytes, and microglia from progenitor cells, which are necessary for maintaining the function of the nervous system [11]. In contrast to neurons, glial cells might easily proliferate in the adult brain. Glial cells actively participate in the function of the central nervous system, affecting metabolism and neurotransmission. Neurotransmitter receptors are present in different types of glial cells [12]. Astrocytes are particularly important for synaptic stability and regulation of the synaptic plasticity [13,14]. Moreover, they participate in glutamatergic transmission and, via reuptake from the synaptic cleft, might modulate the glutamate excitotoxicity [15]. In recent years, neuro- and gliogenesis have been implicated in the pathophysiology of various psychiatric diseases, such as neurodegenerative disorders or affective disorders [16]. Certain drugs, such as selective serotonin reuptake inhibitors or tricyclic antidepressants, were shown to affect gliogenesis [17]. On the other hand, external factors, such as stress, can hinder gliogenesis [17].

As NTs exert a neuroprotective effect, their level will likely be altered by certain challenges, such as hypoxia. One of the most prevalent conditions characterized by a specific form of hypoxia, namely chronic intermittent hypoxia (IH), is obstructive sleep apnea (OSA). It might affect up to 38% of the general adult population, with a higher prevalence in men and people afflicted with immune-mediated diseases [18]. Well-established risk factors for OSA include obesity, greater neck circumference, age, and male sex [18]. The main symptoms of OSA are multiple recurring apneic episodes during sleep due to the collapse of the airways, causing a decrease in oxygen saturation, arousals, and sleep fragmentation. OSA severity level is evaluated during polysomnography (PSG) using the apnea/hypopnea index (AHI)—number of apneas and hypopneas per hour of sleep.

IH carries multiple negative consequences to the organism; these include cardiovascular and metabolic damage as well as the decline in cognitive functions. IH was shown to raise blood pressure (without increasing heart rate) by enhancing the sympathetic stimulation [19]. It also increases oxidative stress via upregulated production of reactive oxygen species, which promotes apoptosis [19]. Moreover, it might impair endothelial function, thus contributing to atherosclerosis [19]. In the liver, IH is thought to contribute to non-alcoholic fatty liver disease, as it was observed to cause inflammation, steatosis, and an increased rate of collagen deposits accumulation [20]. This form of hypoxia in the pancreas is associated with elevated β-cell replication as well as upregulation of insulin production [20]. This effect, associated with type II diabetes, could be enhanced by the overproduction of pro-inflammatory adipokines, such as TNF, C-C motif chemokine ligand 2, and resistin, which contribute to insulin resistance [20]. Since NTs might exert a wide range of effects on both cellular and systemic levels, particularly strongly in the central nervous system, they might be an essential modulator of OSA. The mechanism of this relationship may be based on an IH-dependent response, which triggers oxidative stress and inflammation and, through nuclear factor-κβ (NF-κβ), leads to a decline in NTs expression [21,22]. 

To the best of our knowledge, up to now, three reviews on the subject of the association between OSA and NTs have been published; their authors, however, have focused specifically on BDNF [23,24,25]. In this article, a broader spectrum of neurotrophins is reviewed.

This narrative review aims to summarize the current literature on the role of selected NTs in OSA, as well as their signaling pathways and influence on cellular metabolism. We discuss their role in OSA pathophysiology and possible mechanism through which NTs might modify the disease course, including selected cardiovascular complications related to the impaired nervous system. We also explore potential compensational mechanisms of OSA, which supposedly have a protective role in preserving the cognitive faculties in this group. Moreover, we highlight the role of BDNF in other psychiatric OSA sequelae (e.g., depression).

## 2. Methodology and the Article Scheme

**Figure 2:**
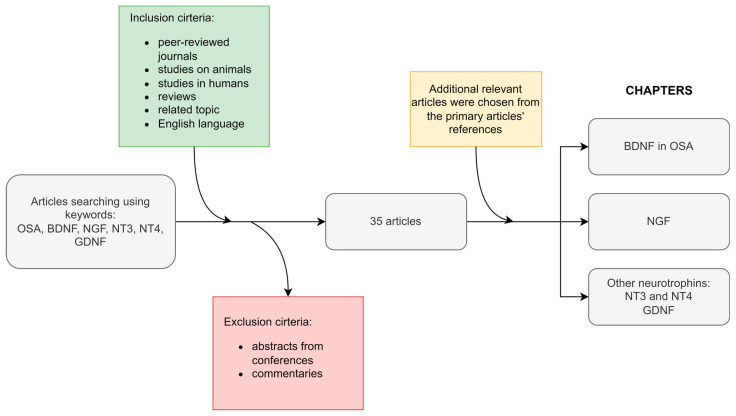
The review article scheme. BDNF—brain-derived neurotrophic factor; GDNF—glial-cell line-derived neurotrophic factor; NGF—nerve growth factor; NT3—neurotrophin-3; NT4—neurotrophin 4; OSA—obstructive sleep apnea.

This article is a narrative review and consists of a critical examination of the literature; Figure 2 depicts the applied methodology in detail. Papers were acquired via PubMed using the following keywords or items in indexed fields: obstructive sleep apnea, BDNF, NGF, NT3, NT4, and GDNF. The inclusion criteria included being published in peer-reviewed journals, studies on animals, studies in humans, reviews on the related topic, and English language of the text. The exclusion criteria were abstracts from conferences and commentaries. Additional relevant articles were chosen from the primary articles’ references. The majority of discussed studies had a cross-sectional design.

## 3. BDNF in OSA

**Figure 3:**
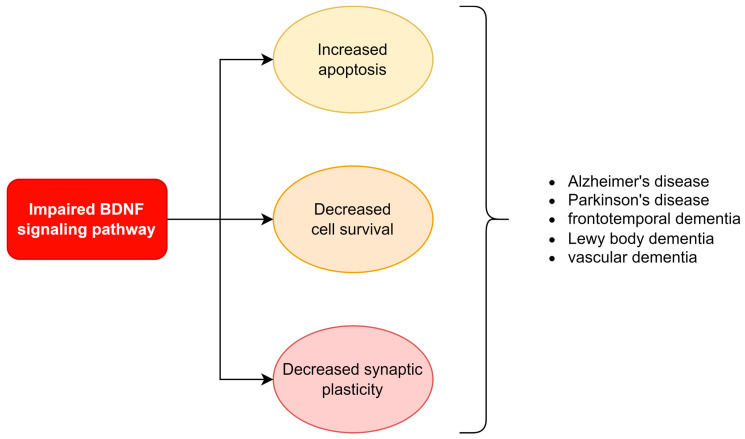
Summary of basic mechanisms through impaired signaling pathway involved in the development of chosen diseases.

BDNF is an NT vital to developing neurons of the central and peripheral nervous system and synaptic plasticity. It has a significant role in memory formation and learning; thus, its expression is often altered in physiological-aging-related or pathological processes affecting cognitive faculties (Figure 3.). The latter group, besides Alzheimer’s disease (AD), includes dementia in the course of Parkinson’s disease and other types of dementia (frontotemporal dementia, Lewy body dementia, vascular dementia) [26,27,28].

BDNF precursor protein, proBDNF, exerts opposite effects to its mature form. Its cognate receptor, p75NTR, might promote apoptosis and neurodegeneration, contrary to TrkB. Thus, the BDNF pathway is vital for regulating different aspects of cell metabolism, such as circadian rhythm or survival [29,30]. The latter occurs by binding BDNF with the TrkB, which recruits the PI3K/Akt pathway [31]. Akt antagonizes pro-apoptotic factors and precludes the expression of the Fas ligand, which affects the inhibition of apoptosis [31]. Due to the high permeability of the blood–brain barrier for this protein, its effects are not confined to the central nervous system.

BDNF might play an important role in the development of cardiovascular OSA complications, such as hypertension and atherosclerosis, or aid in recovery from an ischemic injury. Studies on mice show that BDNF is associated with cardiomyocyte contractility: in one study, perfusion of an animal heart with BDNF enhanced contractile force [32]. Knock-out of both BDNF and tyrosine kinase-inactive isoform of TrkB receptor, prevalent in the myocardium, produced cardiomyopathy [32]. Moreover, TrkB agonists might cause systolic and diastolic reversible hypertensive effects [32]. In the case of atherosclerosis, it was proven that TrkB haplodeficient mice are less prone to developing atherosclerotic plaques on a cholesterol-rich diet [32]. Interestingly, BDNF knock-out in epithelial cells did not influence lesions [32]. Transient ischemia is also followed by a rapid, local increase in BDNF and its immature form onsite [32]. Elevated plasma BDNF has also been observed. Authors suggest that such an effect might play a role in innervation [32]. During embryogenesis, enhanced expression of this NT was shown to increase vascularization of the heart; however, in the adult specimen, this effect is stunted due to a different TrkB isoform as a dominant receptor [32]. Unfortunately, there are no studies directly reporting the effects of BDNF on the cardiovascular system in OSA patients.

BDNF might also slow down the weakening of the soft palate muscles, thus potentially reducing episodes of hypoxia. As one study shows, BDNF expression was upregulated in samples from the uvula of OSA patients, which could contribute to improving mechanically (e.g., due to vibration) damaged nerve fibers [33]. BDNF might also affect the patency of airways through modulation of neuroplasticity in the hypoglossal nucleus [34]. The mechanism behind it is related to serotonergic signaling [34]. This neurotrophin also regulates the respiratory drive: mice lacking BDNF have severe impairments in respiratory control [35].

OSA features two main factors that could be associated with changes in BDNF expression: IH and sleep fragmentation caused by microarousals. However, it is difficult to separate the contribution of these two to the consequence of this disease. Studies on animal models investigating OSA usually consider only the IH while omitting sleep fragmentation. Studies on people afflicted with OSA also have limited possibilities to distinguish between those two factors. Thus, it needs to be noted that animal studies further discussed in this review might not directly reflect changes seen in humans.

### 3.1. Arousals in OSA and Their Influence on BDNF

One of the most characteristic comorbid disorders of OSA is chronic sleep disturbance (CSD), which is an effect of numerous arousals during sleep conditioned by recurrent breathing pauses. It brings the patient out of sleep homeostasis, often unconsciously, and leads, among others, to excessive daytime sleepiness (EDS) and insomnia symptoms, which are the main reasons for seeking medical help. There is some evidence of the connection between daytime sleepiness and BDNF levels in OSA. Kaminska et al. related EDS with BDNF. They showed that higher serum BDNF levels were related to increased EDS, measured by Epworth Sleepiness Scale (ESS). Similar correlations were also obtained for IL-6. Moreover, after a 6-month follow-up, changes in ESS were still associated with BDNF levels [36]. The same relationship between EDS and BDNF was also observed in another study [37]. 

Other studies also highlight the association between BDNF and other parameters used to assess OSA severity. Flores et al. have shown a positive correlation between the oxygen desaturation index (ODI) and plasma BDNF levels in OSA patients prior to their treatment [38]. However, AHI, a related parameter, is rarely discussed in studies; Wang et al. demonstrated its negative correlation with serum BDNF level [39]. Different study materials could explain this discrepancy.

As already mentioned, the effect of arousals on BDNF in OSA patients is difficult to assess. There is only one study in this field. Sleep and wakefulness are controlled by a neural network named wake-activated neurons (WAN), including noradrenergic, orexinergic, histaminergic, cholinergic, serotoninergic, and dopaminergic neurons [40,41]. Disruption in signaling in one or more of those transmissions may impact attention, vigilance, and the ability to sustain wakefulness. Zhu et al., as the only ones, studied degeneration in arousal neurons in CSD in mice models of OSA. They showed that CSD imparts lasting wake impairments without influencing total sleep time and confirmed the neurodegenerative effect of OSA by reduction of WAN associated with increased oxidative stress, upregulation of tumor necrosis factor α (TNF-α), and suppression of BDNF in neuronal cells [40]. On the other hand, some studies on the post-traumatic stress disorder (PTSD) model showed a positive correlation between arousals and BDNF expression in the medial prefrontal cortex, which might lead to altered behavior [42,43,44].

Those results are evidence of the new way of thinking about sleep disturbances and emphasize the role of neuronal transmission in developing CSD in OSA patients. However, there is a need to further investigate the role of BDNF in its pathophysiology of CSD.

### 3.2. Intermittent Hypoxia in OSA and Its Influence on BDNF

IH remains an active area of research; in recent years, molecular mechanisms behind its neurodegenerative ramifications have been elucidated to some extent, highlighting the role of apoptosis-related neural injury or oxidative stress [25]. BDNF might mediate some of those changes, contributing to the overall decline in cognitive functions and psychiatric OSA sequelae, such as depression [45]. As studies show, depressed individuals tend to have a lower BDNF level than the general population [46]. Since it is estimated that up to 63% of OSA patients might suffer from this illness, BDNF alterations could influence the course of depression in this group. However, as of 2022, there are a few studies on this subject; one shows that BDNF bears no relation to depression in women with moderate or severe OSA [47]. A recent study showed that OSA patients with pronounced symptoms of depression had lower levels of BDNF and proBDNF in the morning compared to OSA individuals without mood disturbances [48,49].

To date, only some studies have evaluated BDNF in OSA patients and the IH model, but the outcomes are inconsistent. The majority of evidence indicates that IH, the main factor in the pathophysiology of OSA, causes a decrease in BDNF in animal models [21,50,51,52,53,54]. Fang et al. studied the influence of IH on the neurodegeneration of the optic nerve in mice models. As a result, they showed decreased BDNF level, which was correlated with tissue plasminogen activator (t-PA) level decline and reduced expression of p-TrkB and p-CREB in retina ganglion tissue [54]. t-PA is a protein involved in the breakdown of blood clots, but it is also responsible for proBDNF cleavage and its maturation to BDNF. Previous studies showed that the t-PA gene is the target for CREB, a transcription factor. Moreover, those changes were reversible after an antioxidant—7,8-dihydroxyflavone (7,8-DHF) administration [54]. After 7,8-DHF application, oxidative stress decreased, and BDNF/TrkB/CREB pathway increased in activity. It emphasizes the role of reactive oxygen species (ROS) in the impairment of BDNF expression. The role of the TrkB/CREB pathway in BDNF signaling was confirmed in mouse and piglet models of the OSA [34]. 

Moreover, studies show that BDNF level is lower in materials sampled from animals subjected to IH [51,52,55,56]. In OSA-induced beagles, lower levels of BDNF in the hippocampus and the prefrontal cortex were associated with neurodegenerative changes, such as decreased length, number of dendrites intersections, and spine loss [52]. However, some authors have observed the opposite BDNF changes; in a study by Vermehren-Schmaedick et al., mice subjected to acute IH had increased BDNF levels in the medulla and the pons [57]. Interestingly, methyl-CpG-binding protein 2 knock-outs showed no significant changes regarding the discussed NT (MECP2) [57]. Loss-of-function mutations of MECP2 are known to cause Rett syndrome, and since patients with this disease often suffer from autonomic breathing abnormalities, a lack of BDNF upregulation might contribute to further exacerbations of respiratory control issues and neurodegeneration [57]. Similarly, rats who underwent IH had upregulated BDNF in the ventral cervical spinal cord [58]. The induction of BDNF’s synthesis was dependent on serotonin. Moreover, this NT was vital for the intensification of respiration following IH through its influence on the phrenic nerve [58]. An increase in both BDNF and TrkB was also seen in rats after IH; the production of both proteins was enhanced in motor nuclei (respiratory and nonrespiratory) [59].

Low levels of BDNF might also impair long-term potentiation (LTP), a process that strengthens synapses leading to upregulated signal transmission [60]. Especially in the hippocampus, LTP is crucial for learning and memory formation; it could be divided into early and late phases [60]. Changes resulting from the early phase are not stable; this stage also does not involve protein synthesis in opposition to the late degree, which has more permanent repercussions and is dependent on protein synthesis [60]. BDNF has been evidenced to promote LTP [51]. In mice, a decrease in early phase LTP in the hippocampus was seen after seven days of IH; changes measured after 14 days were similar [51]. On the other hand, the impact of late LTP was more pronounced in mice that underwent IH for 14 days compared to the seven days, thus showing strongly progressive tendencies [51]. Animals from 7- and 14-day groups also had lower levels of BDNF but not proBDNF in the biopsy materials [51]. This indicates some form of impairment in the process of the BDNF post-translational modifications, perhaps also affecting the balance between cell survival and apoptosis, tipping it towards the latter. Moreover, with administration of BDNF to the brain ventricles during the experiment in the 7-day group, the studied subjects showed normal early LTP, which proves that BDNF might prevent damages caused by IH [51]. 

Thus, IH might affect different metabolic pathways associated with cognitive functions, one of which is iron metabolism. Hypobaric IH decreased hepcidin expression in obese mice via upregulation of erythropoietin and downregulation of interleukin 6 [61]. This could be another compensational mechanism activated to preserve cognitive function. In an animal model of IH, hepcidin knock-out mice showed decreased iron deposition via FPN1 degradation, decreased ROS production, elevated BDNF level in the hippocampus, as well as higher synaptic plasticity, which was reflected in comparatively better performance on cognitive tests [62].

BDNF is associated with OSA on many levels; however, most humans studied on BDNF in OSA showed no differences in BDNF levels compared to healthy controls [36,47,48,63,64,65,66]. Discrepancies in this regard are not completely clear; Flores et al. have proposed that sometimes observed increases in BDNF levels might be a compensatory mechanism to offset damages caused by chronic IH [38]. Indeed, in their study, a higher level of BDNF has been associated with better cognitive functioning, as measured by an appropriate questionnaire, the Montreal Cognitive Assessment [38]. The mechanism behind this group’s upregulation of blood BDNF is unclear. Since BDNF might cross the blood–brain barrier, this elevation on the periphery could reflect an increase within the central nervous system (CNS). However, it is necessary to note that vast differences between brain and peripheral levels of BDNF were also observed [67]. Association between cognitive abilities and BDFN reported by Flores et al. strongly suggest the former, i.e., BDNF in the periphery reflects BDNF in the CNS.

A possible explanation of this paradox may also be the time of exposure to IH. In all discussed studies on animal models, the time of IH exposure ranged from 3 to 12 weeks. OSA is a chronic disorder that can remain undiagnosed for many years; moreover, treatment is based on symptom management rather than striving for a full recovery. This time is sufficient to activate certain compensational mechanisms.

The impact of hypoxia-inducible factor 1 (HIF-1) is worth considering in the context of adaptation to IH. As it was recently demonstrated in several studies, OSA patients have increased expression of the α subunit of this protein [66,68,69]. This transcription factor regulates the expression of multiple genes responsible for homeostasis and adaptation to a hypoxic environment, such as erythropoietin, glucose transporters, etc. [70]. Helan et al. observed that inhibition of HIF-1 hinders the effects of hypoxia on the BDNF [71]. BDNF might also induce the expression of HIF-1, which indicates a positive feedback loop between the two [71]. Gao et al. have confirmed this finding; in retinoblastoma cells, BDNF/TrkB induced HIF-1, which additionally weakened the effect of the applied chemotherapeutics [72]. Moreover, BDNF was demonstrated to induce mitophagy, a degradation of mitochondria in the mechanism of autophagy in brain microvascular endothelial cells, through HIF-1/BCL2/adenovirus E1B 19 kDa protein-interacting protein 3 (BNIP3) [73]. This is a protective mechanism in hyperglycemic conditions activated in order to avoid injury [73]. Since OSA is a risk factor for DM2, interactions between HIF-1 and BNIP3 might be an interesting direction for future studies. BDNF is also able to activate the mammalian target of rapamycin (mTOR) signaling—an important positive regulator of the HIF-1/vascular endothelial growth factor (VEGF) pathway—thus exerting neuroprotective influence after ischemic injury [74,75]. This interaction, like the one mentioned before, rather pertains to possible sequelae of OSA and their possibly mildly different course in OSA patients—in this case, an ischemic injury. Additionally, it has been shown that the HIF-1α protein level was an important proBDNF predictor, which indicated its involvement in protection against hypoxia [66].

BDNF is also crucial to maintaining proper cytoarchitectural structure in the central nervous system by offsetting the damages caused by IH. It is able to prevent cell apoptosis by activating the PI3K/Akt pathway by interacting with the TrkB receptor [76]. As one study shows, in mice that underwent IH, apoptotic changes were detectable early on in the study, even before memory deficits could be spotted [77]. 

As already mentioned, IH is inevitably associated with oxidative stress. IH generates reactive oxygen species (ROS), which damage intracellular structures, alter the molecular structure of lipids, protein, and nucleic acid, and finally lead to apoptosis. Mice exposed to IH showed more significant apoptosis of cortical neurons, which was associated with the overactivation of caspase-3. IH activates certain genes and transcription factors, such as NF-κB [30]. NF-κB and BDNF might mutually influence their production [30]. BDNF promoter sequence might bind NF-κB [30]. On the other hand, TrkB pathway stimulation can induce the production of NF-κB [30]. This transcription factor might promote apoptosis in IH conditions [30]. In rats subjected to intermittent hypoxia, the hippocampal level of BDNF was decreased, while the increase in NF-κB and several other pro-inflammatory mediators was observed. Inhibition of serine/threonine kinase, mTOR, and NF-κB with the use of the rapamycin and ammonium pyrrolidine dithiocarbamate has precluded neuronal damage in the hippocampi of studied animals [21]. This shows that targeting pathways related to BDNF might preserve cognitive functions in OSA patients. 

IH also promotes presynaptic glutamate release, which might have neurotoxic effects, causing apoptosis of neurons [78]. This excitotoxicity can be, to some extent, prevented by BDNF due to its activation of the PI3-K and Ras/MAPK pathways [79]. Thus, a decrease in BDNF due to IH might increase the apoptosis rate, contributing to the degeneration of cytoarchitecture. Moreover, in animal models, IH was also shown to impair the excitability of neurons by lowering the membrane resistance via altering the expressions of ion transporters and channels [51,77]. Thus, IH changes not only the architecture but also the functioning of the neurons on an elementary level.

Inconsistencies between studies on circulating BDNF levels might stem from differences between serum and plasma BDNF. A major portion (about 99%) of this protein is stored in platelets which, in order to release BDNF, must coagulate before centrifugation; thus, difference in clotting time might be crucial to the concentration of obtained proteins [80]. This process does not take place in plasma BDNF protein measurements. Moreover, most authors, except for Polyakova et al., have observed no correlation between plasma and serum BDNF [80]. The amount of BDNF in platelets is also prone to changes depending on factors, such as age or gender, and, in women, even phase of the menstrual cycle [81]. On a more clinical note, drugs that could cause elevated BDNF levels, such as cerebrolysin, are instituted in the treatment of mild cognitive impairment, AD, and other types of dementia, often bringing notable clinical improvements [82]. According to a study performed by Alvarez et al., an increase in BDNF in a group treated with cerebrolysine was concomitant to improved cognitive functioning at 16 weeks of observation [83]. A combination therapy with donepezil prolonged this effect, and further progress was seen at 28 weeks [83]. Those results further highlight the importance of BDNF in cognitive functioning.

### 3.3. The Association between Sleep, Pain, OSA Therapy, and BDNF

BDNF also has complex interactions with sleep structure. In animal models, it was able to regulate rapid eye movement (REM) and non-REM (NREM) sleep duration [84]. About 50% reduction in BDNF level could, among others, lower the number of REM episodes and REM duration while not affecting NREM in any substantial way [85]. On the other hand, a reduction in BDNF due to gene polymorphism is associated with a decline in slow-wave sleep duration and sleep intensity in humans [86]. However, as of now, little is known about specific changes in sleep architecture associated with altered levels of BDNF in OSA patients. Researching this subject could elucidate mechanisms related to some symptoms of OSA, for example, an increase in daytime sleepiness (which also has been positively correlated with BDNF level), as well as allow for comparison with sleep patterns seen in psychiatric conditions associated with OSA (e.g., depression or dementia) [36,87]. 

It has been observed that patients with OSA report greater pain severity compared to the general population [88,89]. BDNF, due to its involvement in nociception, could contribute to this effect. BDNF participates in pain perception on a few levels: first, it might be produced by TrkA-positive nociceptors, which end in the spinal dorsal horn [90,91]. Second, it regulates glutamate production in the spine [90,92]. This excitatory neurotransmitter is crucial to the appropriate conduction of pain signals from the receptors. Studies show that BDNF is primarily associated with chronic pain due to central hypersensitization and inflammation rather than the acute one resulting directly from an injury [93,94]. Central sensitization is a process characterized by hyperalgesia and allodynia due to functional and structural changes in neuronal circuits, such as increasing the efficacy of synaptic transmission, neuronal excitability, alterations in gene transcriptions, as well as post-translational modifications [95,96]. The sensitizing effects of BDNF were particularly well documented in patients with fibromyalgia, where widespread musculoskeletal pain of unknown origins is the main complaint. Levels of BDNF are significantly elevated in this group. Moreover, they were shown to be correlated with decreased pressure pain threshold (pain in fibromyalgia can be triggered by applying pressure to the tender points on the body) [97,98]. It is important to mention that level of BDNF is not a single determinant of how severely individuals rate their pain; increased sensitivity to nociceptive stimuli might also occur in conditions where BDNF is decreased, such as insomnia [99,100]. 

Another interesting aspect of the role of BDNF in OSA is its relation to therapy. In a study conducted by Staats et al. on patients with OSAS (OSA, which features pronounced excessive daytime sleepiness), there was no difference in serum or plasma BDNF levels between healthy controls and the OSAS group [101]. The first night of continuous positive air pressure (CPAP) treatment significantly decreased BDNF in peripheral blood. However, after three months of CPAP therapy, BDNF concentrations, although significantly higher, did not return to the values observed before treatment. Interestingly, BDNF secretion was not affected at any timepoint [101]. Authors concluded that this indicates increased neuronal uptake of BDNF for neuroprotective purposes [101]. This explanation, however, contradicts the ability of BDNF to cross the blood–brain barrier unimpededly; further studies on the subject are warranted to explain this phenomenon [101]. In a recent study by Flores et al., a non-significant reduction in serum BDNF in OSA patients was observed [38].

### 3.4. Summary—BDNF in OSA

In summary, BDNF might contribute to the pathophysiology of OSA and its sequelae, particularly those in the area of psychiatry. It shapes the cytoarchitecture as well as the functioning of the nervous system. Since the burden and prevalence of OSA are high in modern societies, new forms of therapy are needed to improve the lives of patients afflicted with this disorder. BDNF appears promising for future studies as it targets burdensome consequences of OSA, which are the decline in cognitive functions and disrupted sleep.

## 4. NGF

NGF is a neurotrophic factor responsible for sympathetic neurons’ differentiation, maturation, and survival. Similarly to BDNF, its primary receptor is TrkA—tyrosine kinase—which acts by Ras/MAPK and PI3K/Akt pathways. NGF’s precursor protein (proNGF) has opposite activity and shows affinity to p75NTR, a receptor belonging to the tumor necrosis factor receptors superfamily [102]. It can bind all neurotrophins and their premature forms [102]. Activating p75NTR might have numerous, sometimes opposite consequences; for example, it has been shown to promote both apoptosis and survival through different molecular pathways [102]. Apart from cross-talk with other neurotrophin receptors, p75NTR might interact with the Nogo-66 receptor, thus regulating the axonal elongation [102]. Moreover, this transmembrane receptor could also be associated with the sortilin receptor. Sortilin-p75NTR complex is necessary for pro-forms of neurotrophins to exert their pro-apoptotic influence [103]. 

Only a few studies investigate the role of NGF in OSA. The outcomes are inconsistent. 

OSA has numerous cardiovascular sequelae. They include hypertension, heart failure, or coronary artery disease [104]. Recently, a strong association between the atrial autonomic nervous system and OSA-induced atrial fibrillation (AF) was found [105]. NGF might contribute to such autonomic nervous system disturbances. Xiaokereti et al. confirmed that in a mouse model, IH enhanced the sympathetic activity of the left stellate ganglion (LSG), which led to neuronal remodeling in LSG and the left atrium. NGF and Fos were upregulated in both locations [106]. Authors used those to assess neural remodeling and innervation but did not propose a possible mechanism behind higher NGF levels. Huan et al. showed that sympathetic stimulation could cause the overactivation of macrophages and increase IL-1β synthesis, a regulator of NGF expression [107,108]. Notably, chronic inflammation, an inseparable element of OSA, may increase the intensity of the process mentioned above [22]. A possible treatment of AF may be the ablation of one of the ganglia of the cardiac autonomic nervous system. It reduced sympathovagal hyperactivity and decreased NGF expression in a canine model of the OSA [109]. In another study, researchers also confirmed sympathetic-derived overexpression of NGF in the same model, which ascertained it as a reciprocal process with pulmonary remodeling [110]. Similarly to BDNF, NGF was found to be increased in cardiac tissues following ischemic injury, which indicates its role in the recovery [32]. 

NGF might also be implicated in the pathophysiology of neurogenic inflammation. In the pediatric population, the most critical contributor to the development of OSA is tonsillar hypertrophy. Goldbart et al. found increased expression of NGF and its receptor, TrkA, in adenotonsillar tissue in pediatric patients with OSA [111]. In the authors’ opinion, the cause is the respiratory syncytial virus-initiated inflammation [111]. Changes in innervation caused by the increase in NGF could modulate the course of infections and exaggerate the immune response to infections [111]. Interestingly, there were no significant changes in levels of other studied neurotrophins in this population, namely NT3 and BDNF [111]. It would be interesting to study whether changes in NGF in children caused by viral infection influence the risk of developing OSA or immune-mediated diseases affecting the respiratory tract later in life.

To some extent, ischemia models might reflect changes seen in IH. Studies on muscle ischemia caused by femoral artery occlusion show increased NGF levels, which probably stimulate the expression of the muscle metaboreceptors in the dorsal root ganglion (DRG) neurons of rats [112]. Moreover, HIF-1 alpha and NGF levels had very similar time courses [112]. OSA patients also are characterized by increased HIF-1 alpha levels [113,114]. NGF expression may probably increase in OSA patients in the same way, but there are no available studies, so it requires further investigation. Interestingly, another study showed that increased NGF mRNA expression is associated with a declined memory performance [115].

On the other hand, there is also research showing that patients with OSA may have reduced or similar NGF levels. Shah et al. did not find any differences in NGF levels in muscle fibers of the uvula in mature patients with and without OSA, opposite to BDNF levels [33]. Silva Junior’s study showed significantly lower serum NGF levels in adolescents with poor sleep quality and excessive daytime sleepiness (EDS) [116]. Sleep disorders other than OSA diagnosed using PSQI-BR in adolescents were also associated with lower NGF levels [116]. EDS seems to be associated with reduced NGF, which is reflected by decreased cognitive performance [117]. 

To summarize, the role of NGF in OSA is unclear, and the literature on this subject is sparse. Similarly to other neurotrophins, it might prevent damage ensuing as a result of IH. However, there are few studies investigating that. Current research reveals its involvement in cardiovascular sequelae of the OSA, related to the dysfunction of the autonomic nervous system. Alterations in NGF levels caused by sleep disturbances might be reflected in their clinical manifestations, e.g., affecting cognitive performance or sleepiness. Available studies on this subject were performed on adolescents; thus, observed changes might differ from those seen in adults, who constitute the vast majority of OSA patients.

## 5. Other Neurotrophins

### 5.1. NT3 and NT4

NT3 and NT4 are proteins with similar properties and functions to those previously discussed in NGF and BDNF. NT3 appears to be the most versatile of them all as it displays affinity to different receptors, primarily TrkC but also p75NTR, TrkB, and TrkA (albeit in a different way than its actual ligand, NGF) [118]. NT4, on the other hand, activates p75NTR and TrkB, thus remaining largely redundant to BDNF [118].

As of 2022, there are no studies on NT3 and NT4 in OSA. Those investigating their role in related areas, i.e., sleep and hypoxia, are also heavily limited. 

One study has shown that intracerebroventricular administration of NT3 and NT4 to rabbits at night increased the amount of time spent in NREM by increasing the number of NREM episodes, without substantially altering the NREM structure [119]. Only a four times higher dose of NT4 was able to decrease the slow-wave activity [119]. Interestingly, the same doses of NTs injected during the day did not impact either the sleep structure or the behavior of animals in the study group [119]. NT4 does not seem to affect the respiratory drive altogether; animals who congenitally lacked this neurotrophin did not show abnormalities in this aspect [35].

Those results indicate mutual interactions between circadian rhythm and mentioned NTs, warranting further studies on this subject. More insight into this relation would help to elucidate changes in sleep structure observed in OSA.

### 5.2. GDNF

GDNF is a protein belonging to the GDNF family of ligands (GFL) together with neurturin, artemin, and persephin. Traditionally it is considered a neurotrophic factor rather than a canonical NT. It has multiple sources within the central nervous system and on the periphery (e.g., astrocytes, motor neurons, or muscles). It is essential to the proper development of the dopaminergic system, promoting the differentiation of dopaminergic neurons. 

Moreover, it is necessary for the formation of the respiratory pattern generator. One study on mice has shown that GDNF knock-out animals had reduced inhibitory input to the central pattern generator, thus increasing breathing frequency [120]. The decrease in inhibitory signals stemmed from an abnormally low number of tyrosine hydroxylase neurons in the rostral ventrolateral pons (group A5) [120]. Mutations of the GDNF gene have also been implicated in the pathogenesis of the central congenital hypoventilation syndrome, further corroborating its significance for regulating respiration [121].

Moreover, as chemo-afferent neurons and chemoreceptive cells type I in the carotid body are dopaminergic, GDNF participates in their development [122]. Therefore, abnormal GDNF expression might impair the function of the carotid body, compromising a respiratory response to hypoxia [122].

Thus, GDNF is suspected to play an important role in the pathogenesis of OSA, accounting for a high degree of heritability of this condition [123]. Cao et al. showed that OSA patients have a lower GDNF expression level than the general population [124]. This observation was replicated by Yuanyuan et al. Their study further reported an association between GDNF and OSA independent of obesity, one of the crucial risk factors of the disorder [125]. Moreover, their research evaluated the potential of GDNF for being a biomarker for OSA, achieving spectacular results with specificity and sensitivity of 100% and an area under the curve equal to 1.00 [125]. The authors did not discuss the potential limitations of these results, yet they should be interpreted with great caution. Larkin et al. have shown that variants of GDNF influence the risk of OSA independently of obesity in a population of European Americans [124]. Moreover, studied single nucleotide polymorphisms (SNP) in the GDNF gene were also associated with the AHI [124]. However, a similar study performed on the Icelandic population showed no relation between GDNF SNPs and AHI or OSA [122].

In summary, GDNF might predispose to OSA development, influencing the formation of structures responsible for the regulation of respiration as well as chemoreception and response to hypoxia. Due to different populations and environments, studies on the subject of SNPs might not yield replicable results. However, due to the potentially high diagnostic value of GDNF gene mutations, further research is warranted as it might allow for earlier onset of treatment, preventing the deleterious consequences of OSA.

## 6. Conclusions

The main findings of the study were summarized in Table 1. NTs appear to have a vital and complex role in the pathophysiology of OSA and its various sequelae. However, apart from BDNF, this relation remains largely under-investigated. Available studies indicate the involvement of some of the NTs in sleep and circadian rhythm disruptions, which might directly reflect clinical manifestations of this condition, e.g., a decrease in sleep quality or excessive daytime sleepiness. They might also affect the disease course and severity of OSA sequelae; BDNF, in particular, has been strongly associated with cognitive decline, frequently seen in those patients later in the disease course. On the other hand, NGF might modulate the autonomic nervous system’s dysfunction, strongly influencing cardiovascular pathologies seen in OSA patients. NT3 and NT4 are the least studied NTs. However, they seem to display a particularly strong connection to the circadian rhythm, which OSA could disrupt due to sleep fragmentation. GDNF, in contrast to other discussed proteins, could be considered an essential factor in the pathogenesis of this condition, as it affects the formation of the respiratory system and chemoreception, thus modulating response to hypoxia. Further research on the subject of NTs would increase knowledge of the OSA pathophysiology, which could help in the search for new treatment targets suited to patients’ individual disease profiles.

## Figures and Tables

**Table 1:** Summary of the main findings from the review.

Summary of Main Findings
BDNF is the neurotrophin that is most extensively described in the context of OSA pathophysiology. The altered BDNF signaling pathway has an impact on the development of OSA sequela, including cardiovascular diseases and especially psychiatric disorders, including chronic sleep disturbance, cognitive impairment, and depression. Available data suggest that BDNF may be the marker of CPAP treatment effectiveness and might be used to predict the possible development of psychiatric sequela. Recent studies indicate NGF as a plausible prediction factor of OSA-induced AF development. More studies are needed to evaluate the involvement of all neurotrophins in the course and complications of OSA to fully comprehend the spectrum of their effect.

AF—atrial fibrillation; BDNF—brain-derived neurotrophic factor; CPAP—continuous positive airway pressure treatment; NGF—nerve growth factor; OSA—obstructive sleep apnea.

## Data Availability

Not applicable.
